# P01-014 – Subclinical atherosclerosis in FMF

**DOI:** 10.1186/1546-0096-11-S1-A18

**Published:** 2013-11-08

**Authors:** S Ugurlu, SN Karaca, Y Demirel, E Seyahi

**Affiliations:** 1 Department of Internal Medicine, Division of Rheumatology, Cerrahpasa Medical Faculty, University of Istanbul, Istanbul, Turkey; 2 Department of Family Mediciney, Medical Faculty, University of Cumhuriyet, Sivas, Turkey, Sivas, Turkey

## Introduction

Whether atherosclerosis is increased or not in Familial Mediterranean Fever (FMF) is a much debated issue. Carotid artery intima media thickness (IMT) a surrogate marker for subclinical atherosclerosis is found to be increased in a number of studies [1-5], however this contrasts with the lack of increased frequency of atherosclerotic plaques [1-5]. Also, population surveys do not indicate an increased prevalence of ischaemic heart disease in FMF patients. In the current study, we hypothesized that FMF patients with no apparent atherosclerotic risk factor would have no increase in the carotid artery IMT.

## Objectives

To investigate markers of carotid atherosclerosis and oxidized low density lipoprotein (oxLDL) levels in patients with FMF who have no risk factors for cardiovascular disease.

## Methods

We studied 44 patients (25 F/19 M; mean age: 33.5±7.5) with FMF in attack free period and gender and age matched 44 healthy subjects (25 F/19 M; mean age: 33.4±7.0). Exclusion criteria were clinical coronary artery disease, chronic renal disease, diabetes mellitus, hypertension, history of myocardial infarction, angina pectoris or cerebrovascular disease, dyslipidemia, metabolic syndrome or active infection. Those who were in postmenopausal status and using antilipid drugs were also excluded. We measured carotid artery IMT and investigated presence or absence of atherosclerotic plaques in the carotids, using Doppler ultrasound. We also assessed serum lipid and OxLDL levels.

## Results

Mean disease duration of the FMF patients was 20±9 years. The mean carotid IMT (C-IMT) did not differ between patients and controls (0.52±0.10 mm vs 0.53±0.06 mm, respectively, P=0.709). None of the patients or controls had atherosclerotic plaques. Total and LDL cholesterol levels were significantly lower among patients compared to controls (total cholesterol: 157.07±34.18 vs 181.05±36.79, respectively, P=0.002; LDL cholesterol: 100.48±30.13 vs 126.25±34.05, respectively, P=0.001). Whereas OxLDL levels were significantly higher in FMF patients (337.48±438.56 ng/dl) when compared to controls (156.19±383.24 ng/dl), (P=0.044). There was no correlation between CIMT and OxLDL levels among both patients (r= -0.156, p=0.324) and controls (r=-0.196, p=0.246).

## Conclusion

Our study supports further evidence for no increased atherosclerosis in FMF. As previously shown patients with FMF have low cholesterol levels when compared to healthy controls [1,2]. On the other hand, increased OxLDL levels could be associated with increased sub-clinic inflammation.

## Disclosure of interest

None declared.
